# Stromal Galectin-1 Promotes Colorectal Cancer Cancer-Initiating Cell Features and Disease Dissemination Through SOX9 and β-Catenin: Development of Niche-Based Biomarkers

**DOI:** 10.3389/fonc.2021.716055

**Published:** 2021-09-10

**Authors:** Kai-Yen Peng, Shih-Sheng Jiang, Yu-Wei Lee, Fang-Yu Tsai, Chia-Chi Chang, Li-Tzong Chen, B. Linju Yen

**Affiliations:** ^1^Regenerative Medicine Research Group, Institute of Cellular & System Medicine, National Health Research Institutes (NHRI), Zhunan, Taiwan; ^2^National Institute of Cancer Research, NHRI, Zhunan, Taiwan; ^3^Graduate Institute of Life Sciences, National Defense Medical Center, Taipei, Taiwan; ^4^Department of Oncology, National Cheng Kung University Hospital, College of Medicine, National Cheng Kung University, Tainan, Taiwan; ^5^Division of Hematology/Oncology, Department of Internal Medicine, Kaohsiung Medical University Hospital, Kaohsiung, Taiwan

**Keywords:** cancer-initiating cells (CICs), tumor stroma, galectin-1, SOX9, biomarkers

## Abstract

Over 90% of colorectal cancer (CRC) patients have mutations in the Wnt/β-catenin pathway, making the development of biomarkers difficult based on this critical oncogenic pathway. Recent studies demonstrate that CRC tumor niche-stromal cells can activate β-catenin in cancer-initiating cells (CICs), leading to disease progression. We therefore sought to elucidate the molecular interactions between stromal and CRC cells for the development of prognostically relevant biomarkers. Assessment of CIC induction and β-catenin activation in CRC cells with two human fibroblast cell-conditioned medium (CM) was performed with subsequent mass spectrometry (MS) analysis to identify the potential paracrine factors. *In vitro* assessment with the identified factor and *in vivo* validation using two mouse models of disease dissemination and metastasis was performed. Prediction of additional molecular players with Ingenuity pathway analysis was performed, with subsequent *in vitro* and translational validation using human CRC tissue microarray and multiple transcriptome databases for analysis. We found that fibroblast-CM significantly enhanced multiple CIC properties including sphere formation, β-catenin activation, and drug resistance in CRC cells. MS identified galectin-1 (Gal-1) to be the secreted factor and Gal-1 alone was sufficient to induce multiple CIC properties *in vitro* and disease progression in both mouse models. IPA predicted SOX9 to be involved in the Gal-1/β-catenin interactions, which was validated *in vitro*, with Gal-1 and/or SOX9—particularly Gal-1^high^/SOX9^high^ samples—significantly correlating with multiple aspects of clinical disease progression. Stromal-secreted Gal-1 promotes CIC-features and disease dissemination in CRC through SOX9 and β-catenin, with Gal-1 and SOX9 having a strong clinical prognostic value.

## Introduction

Colorectal cancer (CRC) is one of most common cancers worldwide, with its incidence and mortality rising in individuals age 50 and younger (1). While overall decreases in the CRC risk factors of alcohol intake and smoking along with an increased screening has helped reduce its incidence for several decades, alarmingly, in recent years, incidence rates have been on the increase in younger adults 50 years and under (2, 3). Survival rates of CRC strongly correlate with stage, with the 5-year survival for stage I or localized disease (excluding carcinoma-*in-situ*) close to 90%, but decreasing to less than 20% for stage IV or metastatic disease (4, 5). Thus, understanding of the specific molecular factors involved in CRC metastases is important for the control of this globally prevalent cancer.

The Wnt/β-catenin pathway is central to CRC, with the initial step in carcinogenesis determined to be mutations in the *adenomatous polyposis coli* gene, which then results in the activation of β-catenin, a transcription factor critical in the maintenance of the normal intestinal stem cell compartment as well as the cells-of-origin or carcinoma-initiating cells (CICs) for CRC (6–8). Over 90% of patients have mutations in the Wnt/β-catenin pathway, making components of this pathway ironically not useful as prognostic markers (9); rather, the functional triggering of the pathway appears to be a more robust evidence for disease progression (10). Paracrine factors secreted by non-cancerous cells within the tumor microenvironment including stromal cells play important roles in the tumorigenesis of CRC. Fibroblasts, which are key components of the stroma, can promote tumorigenic and metastatic capacity in CRC CICs through the upregulation of β-catenin activity (11–13). Despite such recent key findings, there has not been much effort in using the tumor niche to search for prognostic biomarkers. We therefore became interested in elucidating the molecular interactions between tumor niche stromal cells and CRC metastasis, and to develop prognostically relevant biomarkers based on these interactions.

In this study, we determined the interactions of fibroblast-secreted factors on CRC progression, and found galectin-1 (Gal-1) to be highly secreted by two lines of human fibroblasts as determined by mass spectrometry (MS) analyses of the fibroblast-conditioned medium (CM). Gal-1 is a glycoprotein encoded by the *LGALS1* gene and known to exert immunomodulatory effects including mediating tumor-immune escape (14). We found that the secreted Gal-1 has prominent direct tumor-promoting effects in CRC including enhancing CIC features and β-catenin activity *in vitro*, as well as *in vivo* tumor dissemination and disease progression. Moreover, as predicted by the Ingenuity Pathway Analysis (IPA), we validated the involvement of SOX9 (15)—a newly identified marker for aggressive CRC based on a recent large-scale integrative analysis—in Gal-1/β-catenin interactions. Analyses using human CRC transcriptomic databases and immunohistological staining of tissue array corroborated the strong clinical relevance of Gal-1 and SOX9—particularly Gal-1^high^/SOX9^high^ samples—as significantly and prognostically correlated with disease presence and progression.

## Materials and Methods

### Cell Culture

The human CRC cell lines KM12C was obtained from the Korean Cell Line Bank (catalog no.: 80015) (16, 17). The cells were cultured as recommended in a complete medium consisting of Dulbecco’s Modified Eagle Medium: Nutrient Mixture F-12 (DMEM/F-12) supplemented with 10% fetal bovine serum (FBS), 2 mM L-glutamine, and 100 U/ml penicillin-streptomycin (all from Invitrogen-Thermo Fisher Scientific, Waltham, MA, USA). The human fibroblast cell lines MRC-5, derived from fetal lung tissue, and WS1, derived from fetal midscapular skin, were obtained from the Bioresource Collection and Research Center (BCRC, Hsinchu, Taiwan) and were cultured as recommended in the Minimum Essential Medium (MEM) with 10% FBS, 2 mM L-glutamine, and 100 U/ml penicillin-streptomycin (all from Invitrogen). Conditioned medium (CM) was collected from the cell culture after 48 hours of culturing. All cell lines were authenticated using a short-tandem repeat profiling.

### Invasion Assay

Cells were treated with mitomycin C (10 ug/ml) for 2 hours to inhibit proliferation, and then 1x10^5^ cells were seeded on Matrigel-coated chambers containing 75% Matrigel (Sigma-Aldrich, MO, USA; plates with 8.0-μm pores, BD Bioscience, Franklin Lakes, NJ, USA). After CM or treatment with recombinant protein for 48 hours, detection by light microscopy (Leica Microsystems, Wetzlar, Germany) for quantification of invading cells in polycarbonate membranes was performed. Each chamber was sampled at nine different sites. Images were quantified for the number of invading cells using the Image J software (National Institutes of Health (NIH), USA).

### Quantitative Real-Time Polymerase Chain Reaction

qPCR was performed as previously reported (18). Briefly, RNA was extracted from cells with TRIzol (Invitrogen), and converted to cDNA with the ImProm-ll Reverse Transcriptase system (Promega, Madison, WI, USA) according to the protocol of the manufacturer. qPCR was carried out with Fast SYBR^®^ Green Master Mix containing Thermo-Start DNA polymerase on the ABI 7500 Real-Time PCR System (both from Applied Biosystems-Thermo Fisher Scientific). Relative gene expression levels were analyzed as indicated by the manufacturer. The specific primers used are shown in **Supplementary Table S1**.

### Western Blot

Western blot was performed as previously reported (18). Cells were collected from a 10-cm^2^ dish (8 x 10^5^ cells/dish) and detected for whole cell or nuclear proteins, which was isolated with the nuclear extraction kit (Millipore-Merck, Darmstadt, Germany) according to the recommendations of the manufacturer. Extracted proteins were separated by electrophoresis on a 12.5% SDS-polyacrylamide gel and transferred to a nitrocellulose membrane. The membranes were blotted with antibodies against β-catenin (1:1,000; Cat. No.610153; BD Bioscience), Gal-1 (1:1,000; Cat. No.437400; Invitrogen), Slug (1:1,000; Cat. No. ABE993; Millipore), E-cadherin (1:1,000; Cat. No. MAB3199; Millipore), SOX9 (1:1,000; Cat. No. ab3697; Abcam), GAPDH (1:8,000; Cat. No.14-9523-80; eBioscience, CA, USA), α-Tubulin (1:8,000; Cat. No.14-4502-82; eBioscience), or Histone H1 (1:1,000; Cat. No. ab125027; Abcam). Detection was performed using horseradish peroxidase (HRP)-conjugated secondary antibodies and chemiluminescent HRP substrate (Millipore).

### Small-Interfering RNA Knockdown Experiments

Gene knockdown experiments were performed as previously reported (18). Lipofectamine RNAiMAX Reagent (Invitrogen) was used to transfect siRNA specific for galectin-1 (*LGALS1*) or a non-target control (Invitrogen) into cells according to the recommendations of the manufacturer. After 48 hours, RT-PCR and Western blot were used to confirm the expression levels of *LGALS1* in transfected cells. For short hairpin RNA (shRNA) knockdown experiments, MISSION TRC shRNA Lentiviral Particles (Sigma-Aldrich) containing *LGALS1* or *SOX9* shRNA or shLuc were used to infect the cells, which were seeded on 24-wells plates (1 x 10^5^ cells/well) for 48 hours. The infected cells were treated with puromycin (2 ug/ml; Invitrogen) for two weeks to select the stably infected cells.

### Sphere Formation

For sphere formation, cells were seeded in 6-cm^2^ dishes coated with 0.4% agarose gel (6 x 10^5^ cells/well), and grown in serum-free DMEM/F-12 containing 20 ng/ml human recombinant epidermal growth factor (EGF) and 10 ng/ml basic fibroblast growth factor (bFGF; both from Peprotech, Rocky Hill, NJ, USA) for 72 hours (10). For each condition, 15 sites were randomly sampled with light microscopy (Leica Microsystems) and then quantified for the number of spheres (>30 μm) using the Image J software.

### Mass Spectrometric Analysis

MS analysis on the secretome of fibroblasts was performed by Proteomics Core Lab of Chang Gung University (Taoyuan, Taiwan) as previously reported (19). Briefly, CM (24 ml) was harvested from the fibroblasts cultured in a T175 flask for 48 hours. CM was concentrated by centrifugation in Amicon Ultra-15 tubes (molecular weight cutoff 10 KDa, Millipore) five times at 4,000 g for 30 minutes each time. Proteins were separated by 2D gel electrophoresis and subjected to silver staining. Protein bands were extracted and analyzed for peptide mass by MS with MS/MS used to analyze CM protein profiles.

### Enzyme Linked Immunosorbent Assay

ELISA was performed as previously reported (20). Briefly, mouse monoclonal anti-Gal-1 antibody (1:500; Cat. No.437400; Invitrogen) was coated in 96-well plates at 4°C overnight. CM was added into the wells for 2 hours at room temperature. After PBST (PBS with 0.1% Tween 20) wash, wells were incubated in biotinylated rabbit anti-Gal-1 antibody (1:2,000; Cat No.500-P210; Peprotech) for 1 hour. Subsequently, HRP-conjugated streptavidin (1:200; R&D systems, MN, USA) and TMB substrate (Invitrogen) were used to detect biotinylated signaling. Finally, the reaction was stopped by 2N H_2_SO_4_ and absorbance was measured by optical density at 450 nm. Recombinant human Gal-1 (Peprotech) was used as a positive control. The detection range of the standard curve was from 0 to 20,000 pg/ml.

### Luciferase Reporter Assay

The β-catenin-mediated transcriptional activation reporter plasmids of TOPFlash and TOPFlash mutant (contains mutated TCF/LEF binding sites) were obtained from Addgene (Cambridge, MA, USA). Reporter plasmids were transfected with renilla reporter plasmids (for internal control) into cells by using Lipofectamine 2000 reagent (Invitrogen). After 48 hours of transfection, Dual-Luciferase Reporter System (Promega) was used to measure the luciferase activity.

### Immunofluorescence Staining

IF was performed as previously reported (21). Briefly, cells were fixed with 4% paraformaldehyde and permeabilized with 0.1% Triton-X 100 (Sigma-Aldrich) for 20 minutes. After blocking, cells were incubated with primary antibodies against E-cadherin (1:100; Cat. No. MAB3199; Millipore) and β-catenin (1:100; Cat. No.610153; BD Biosciences) at room temperature for 2 hours, and then incubated for 1 hour with FITC-conjugated secondary antibodies (1:200; Santa Cruz Biotechnology, Santa Cruz, CA, USA). Nuclei were stained with Hoechst 33342 (Sigma-Aldrich) and cells were visualized by fluorescence microscopy (Olympus, Tokyo, Japan).

### Drug Resistance Assay

Cells were seeded in a 24-well plate (4 x 10^4^ cells/plate) and pretreated with CM (1:1 mixed with culture medium) or human Gal-1 recombinant protein (rhGal1) for 24 hours. Cisplatin (25 µM; Sigma-Aldrich) was then added to the plates with assessment for cell viability after 48 hours using the 3-(4,5-Dimethyl-2-thiazolyl)-2,5-diphenyl-2H-tetrazolium bromide (MTT) assay (Sigma-Aldrich).

### Ingenuity Pathway Analysis (IPA)

IPA (QIAGEN, Redwood, CA, USA) was used to infer the potential pathways in CRC disease progression involving *LGALS1*, *CTNNB1*, and *Twist1*. The pathway explorer of IPA was used to analyze the direct and indirect interactions of these three genes by utilizing Ingenuity Pathways Knowledge Base.

### *In Vivo* Tumor Dissemination and Metastases Experiments

Animal experimentation was performed in accordance with protocols approved by the Institutional Animal Care and Use Committee (approval number: 1080102). All animals were obtained from the National Laboratory Animal Center of Taiwan (Taipei, Taiwan). A rapid metastatic tumor dissemination study was performed (22). WS1 fibroblasts and KM12C CRC cells were labeled respectively with 5 μM of the fluorescent cellular dyes 1,1’-Dioctadecyl-3,3,3’,3’-Tetramethylindocarbocyanine Perchlorate (DiI) and 3,3’-Dioctadecyloxacarbocyanine Perchlorate (DiO) (both from Invitrogen) for 5 minutes. WS1 (3 x 10^5^ cells) were co-cultured with KM12C (3 x 10^5^ cells) in a 1:1 ratio for 24 hours and injected into the tail vein of C57BL/6 mice (6–8 weeks old). Mice were then sacrificed 24 hours after injection. The lungs were extracted for sectioning (0.5 mm thickness) with the detection of labeled cells with fluorescence microscopy (Olympus). The fluorescence intensity of images was measured by the Image J software. For *in vivo* metastatic experiments, KM12C (3 x 10^5^ cells) co-cultured with WS1 silenced for short-hairpin RNA (shRNA) of non-target sequences (shC-WS1; 3 x 10^5^ cells) or with WS1silenced with shRNA specific for Gal-1 (shGal-WS1; 3 x 10^5^ cells) for one day, then injected into the tail veins of NOD-SCID mice (6–8 weeks old) and followed for up to 6 weeks with weekly measurement of the body weight. Mice were then sacrificed with lung and spleen tissues collected for histological examination.

### Immunohistochemistry of Mouse and Human Tissue

Tissue samples from mice were fixed with 10% formaldehyde and embedded with the optimal cutting temperature (OCT) compound prior to frozen sectioning (Sakura Finetek, Tokyo, Japan). Tissue sections were stained with anti-human histone H1 antibody (1:100; Cat. No. ab125027; Abcam) followed by peroxidase detection (Pierce-Thermo Fisher Scientific) to detect human cells in murine lung and spleen sections. Human CRC tissue arrays were obtained from SUPER BIO CHIPS (Seoul, Korea). The tissue slides were dewaxed with xylene, rehydrated in ethanol, and subsequently stained with antibodies against human Gal-1 (1:100; Cat. No. 437400; Invitrogen) and SOX9 (1:100; Cat. No. AB5535; Millipore).

### Public Microarray Gene Expression Dataset Analyses

CRC transcriptome datasets including GSE33113, GSE17536, and GSE9348 were downloaded from Gene Expression Omnibus (GEO) databases of the National Center for Biotechnology Information (https://www.ncbi.nlm.nih.gov/gds/). The Cancer Genome Atlas (TCGA) database was obtained from the NIH (https://cancergenome.nih.gov). Information on the public gene expression datasets used in this study are listed in **Supplementary Table S2**. GSE33113 and GSE17536 were used to analyze the expression of *CTNNB1* and *LGALS1* (23, 24). For comparing the gene expression levels between normal colon tissues and CRC tissue, Oncomine (http://www.oncomine.org) was utilized to analyze for the expression levels of *LGALS1* and *SOX9* in the Kaiser Colon database (25). To analyze the expression levels of *LGALS1* and *SOX9* with respect to early stages of CRC compared to normal colon tissue, GSE9348 and TCGA were used (26, 27), while GSE17536 and TCGA were used to analyze for the stage-specific expression of *LGALS1*, *SOX9*, *and CTNNB1* (24, 27).

### Statistical Analyses

All experiments were performed at least in triplicate, with data were represented as mean ± SEM. Statistical analyses were performed using the Student’s *t* test for comparisons of two variables and ANOVA for comparisons of more than two variables. For CRC patient transcriptome databases GSE33113, GSE7536, GSE9348, and TCGA, Student’s *t* test was used for the analysis of differences in the specific gene expression levels at each stage of CRC. A value of *p* < 0.05 was defined as statistically significant.

## Results

### Fibroblast-Secreted Factors Significantly Promote Multiple Cancer-Initiating Cell Features in Colorectal Cells

**Figure 1:**
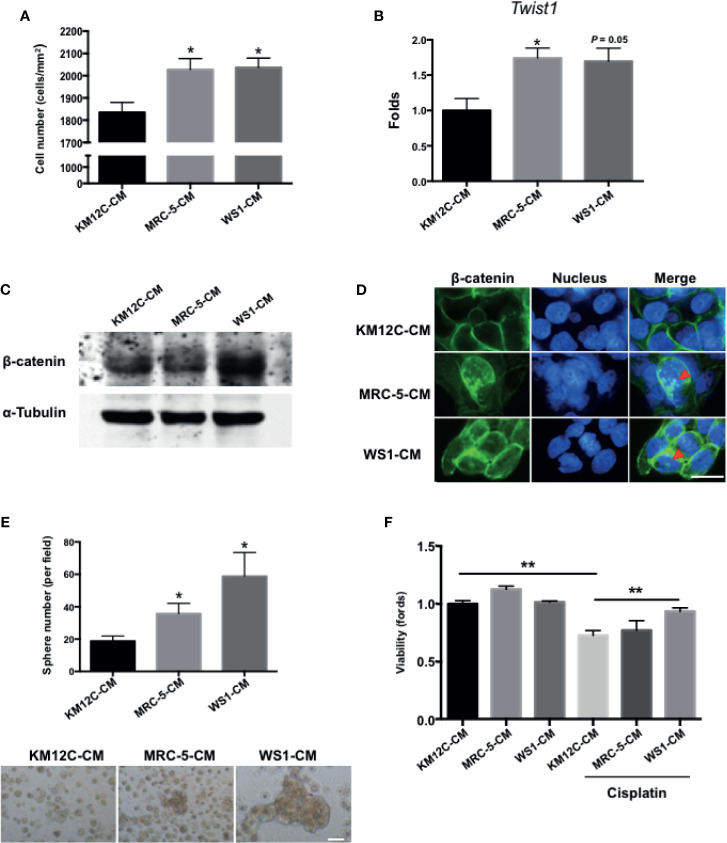
Fibroblast-derived paracrine factors significantly promote multiple cancer-initiating cell (CIC) features in colorectal cancer (CRC) cells. **(A)** Invasion capacity of the CRC cell line KM12C (KM12C) after culturing in conditioned media (CM) of two human fibroblast cell lines MRC-5 and WS1 for 48 hours; control, KM12C-CM. **(B)** Quantitative real-time PCR (qPCR) analysis for the gene expression levels of *Twist1* in KM12C after culturing in MRC-5- or WS1-CM; control, KM12C-CM. **(C)** Western blot for the β-catenin levels in KM12C after culturing in MRC-5- or WS1-CM; control, KM12C-CM. **(D)** Immunofluorescent (IF) staining for β-catenin subcellular localization (green fluorescence) in KM12C after culturing in MRC-5- or WS1-CM; control, KM12C-CM; arrows indicate nuclear β-catenin. Hoechst 33342 was used to detect cell nuclei (blue fluorescence); scale bar, 10 μm. **(E)** Sphere formation capacity of KM12C after culturing in MRC-5- or WS1-CM for 72 hours; control, KM12C-CM. Quantitative results (top panel) and representative images (bottom panel) are shown; scale bar, 30 μm. **(F)** Drug resistance capacity of KM12C to cisplatin (25 µM) after pretreatment with either MRC-5- or WS1-CM (control, KM12C-CM) for 24 hours. Cell viability was assessed 48 hours after drug treatment. All results are shown as mean ± SEM of three independent experiments. *p < 0.05 and ** compared to control.

To assess whether fibroblast-derived paracrine factors are involved in CRC progression, the CRC cell line KM12C was cultured in the CM of two human fibroblast cell lines, MRC-5, and WS1, and assessed for a number of CIC properties including invasive capacity, epithelial-mesenchymal transition (EMT), β-catenin translocation, sphere formation, and drug resistance; these functional assays have been shown to be more relevant to disease progression than CIC markers such as CD133 (28). When cultured in MRC-5- and WS1-CM, the invasive capacity of KM12C was significantly increased (**Figure 1A**) and expression of *Twist1*, a critical transcription factor involved in EMT, was increased significantly up to 2-fold (**Figure 1B**). Moreover, we found that after culturing in either MRC-5- or WS1-CM in particular, β-catenin protein levels in KM12C were increased (**Figure 1C**) with the occurrence of nuclear translocation (**Figure 1D**), which has been reported to enhance CRC tumorigenesis and CIC formation (12). In addition, the sphere formation capacity as well as drug resistance were significantly increased in KM12C after culturing in either MRC-5- or WS1-CM (**Figures 1E, F**). We found that KM12C, which was pretreated with MRC-5- or WS1-CM demonstrated a significantly increased resistance to cisplatin-induced cell death, particularly after WS1-CM pretreatment. MRC-5- and WS1-CM also increased CD29 and CD44 expressions, two well-studied CIC markers, as assessed by flow cytometric analysis (29, 30), in KM12C (**Supplementary Figure S1**). These findings therefore demonstrate that fibroblast-derived paracrine factors significantly promote multiple CIC features in CRC cells.

### Fibroblast-Secreted Gal-1 Significantly Promotes Multiple Cancer-Initiating Cell Features in Colorectal Cancer Cells

**Figure 2:**
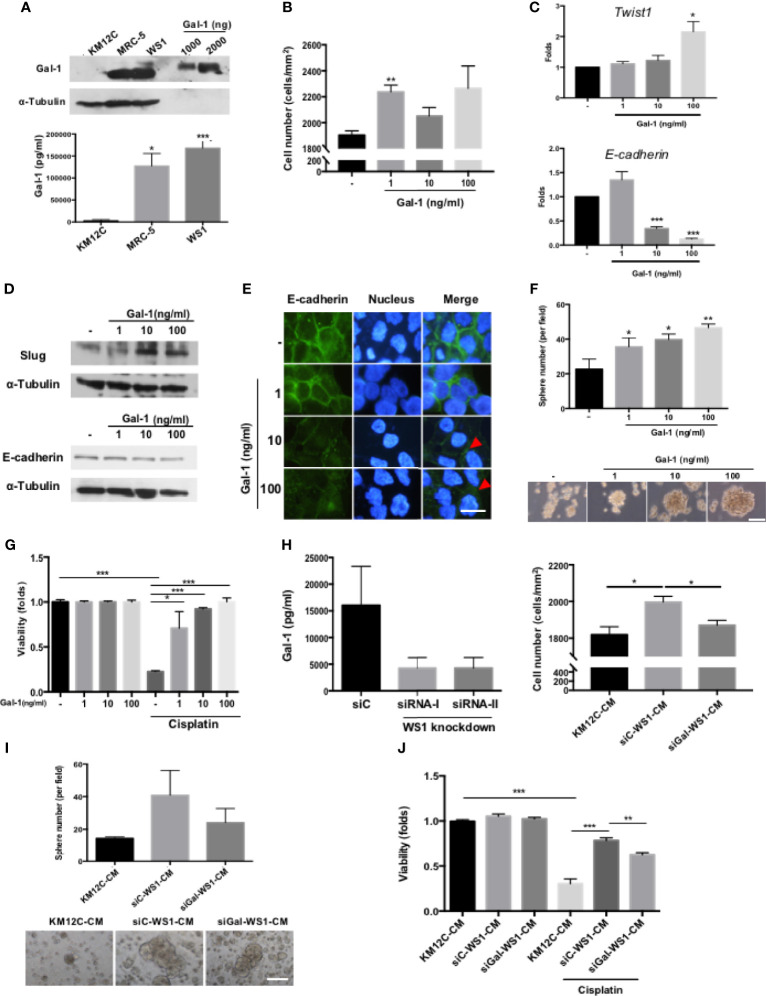
Fibroblast-secreted galectin-1 (Gal-1) significantly promotes multiple CIC features in CRC cells. **(A)** Expression of endogenous Gal-1 in MRC-5 and WS1 fibroblasts as detected through Western blot (top panel; recombinant human Gal-1 protein (rhGal-1) used as positive control) and ELISA (bottom panel). **(B)** Invasion capacity of KM12C with the addition of increasing doses of rhGal-1. **(C)** qPCR analysis for the gene expression of *Twist1* and *E-cadherin* and **(D)** Western blot for protein expression of Slug and E-cadherin in KM12C with the addition of increasing doses of rhGal-1. **(E)** IF staining of E-cadherin (green fluorescence) in KM12C with the addition of increasing doses of rhGal-1 for 48 hours. Nuclei were stained with Hoechst 33342 (blue fluorescence); scale bar, 10 μm. **(F)** Sphere formation capacity of KM12C with the addition of increasing doses of rhGal-1; quantitative results (top panel) and representative images (bottom panel) are shown; scale bar, 30 μm. **(G)** Drug resistance capacity of KM12C to cisplatin (25 µM) after pretreatment with increasing dosages of rhGal-1 for 24 hours. Cell viability was assessed 48 hours after drug treatment. **(H)** Left panel: Validation of Gal-1 knockdown using small interfering RNA (siRNA) specific for Gal-1 (siRNA-I & siRNA-II) in WS1 fibroblasts. Non-target siRNA was used as a negative control. After 48 hours, CM from siRNA-I, siRNA-II, and siC were removed and analyzed by ELISA. Right panel: Invasion capacity of KM12C after culturing in siGal-WS1-CM compared to siC-WS1-CM for 48 hours. **(I)** Sphere formation capacity of KM12C cultured in KM12C-CM, siC-WS1-CM, or siGal-WS1-CM for 72 hours; quantitative results (top panel) and representative images (bottom panel) are shown; scale bar, 30 μm. **(J)** Drug resistance capacity of KM12C to cisplatin (25 µM) after pretreatment with KM12C-CM, siGal-WS1-CM, or siC-WS1-CM for 24 hours. Cell viability was assessed 48 hours after drug treatment; control, KM12C without cisplatin treatment. All results are shown as the mean ± SEM of three independent experiments. *p < 0.05; **p < 0.01, and ***p < 0.005 compared to the control.

To identify the specific fibroblast-derived paracrine factor(s) responsible for enhancing multiple CIC features, MS/MS was used to analyze the secretome of MRC-5- and WS1-CM, and Gal-1 was identified as the most highly secreted protein by both fibroblast populations (**Supplementary Figure S2**), which we confirmed with Western blot as well as ELISA (**Figure 2A**). While Gal-1 (*LGALS-1*) is well known to be involved in cancer immune evasion through modulating specific subpopulations of immune cells, there have been no reports of this protein directly targeting the cancer cell itself to promote CIC features. We therefore assessed whether Gal-1 could be the paracrine factor in fibroblast-CM directly responsible for promoting multiple CIC features in CRC cells. We found that the addition of recombinant human Gal-1 protein (rhGal-1) significantly enhanced the invasive capacity of KM12C (**Figure 2B**). Moreover, the addition of rhGal-1 promoted the EMT of KM12C in a dose-dependent fashion as evidenced by significant increases in the gene expression of *Twist1* with a decreased expression of *E-cadherin* (**Figure 2C**). This was also seen at the protein level with an increased expression of Slug, another transcription factor involved in EMT, along with a concomitant decreased expression of E-cadherin (**Figure 2D**); IF staining also demonstrated a loss of E-cadherin expression at the cell junctions with the addition of rhGal-1 (**Figure 2E**). In addition, the sphere formation capacity (**Figure 2F**) as well as drug resistance (**Figure 2G**) were both significantly enhanced by rhGal-1 in a dose-dependent fashion. Addition of rhGal-1 also increased the expression of CD29 and CD44 in KM12C as well as HCT-116, another well-studied CRC line (**Supplementary Figure S3**). To further verify the role of fibroblast-secreted Gal-1 in promoting CIC features, we generated Gal-1-knockdown WS-1 fibroblasts (siGal-WS1) using gene-specific siRNA (siRNA-I; **Figure 2H**, left panel). We found that KM12C cultured in siGal-WS1-CM demonstrated a significant decreased capacity for invasion, compared to KM12C cultured in control non-target siRNA knockdown WS1-CM (siC; **Figure 2H**, right panel). Correspondingly, KM12C cultured in siGal-WS1-CM compared to siC-WS1-CM also showed a significantly decreased capacity in terms of sphere formation (**Figure 2I**) as well as drug resistance (**Figure 2J**). These results collectively demonstrate that fibroblast-secreted Gal-1 is involved in promoting multiple CIC features of CRC cells.

### Fibroblast-Secreted Gal-1 Significantly Increases Metastases and Tumor Dissemination of Colorectal Cancer Cells *In Vivo*

**Figure 3:**
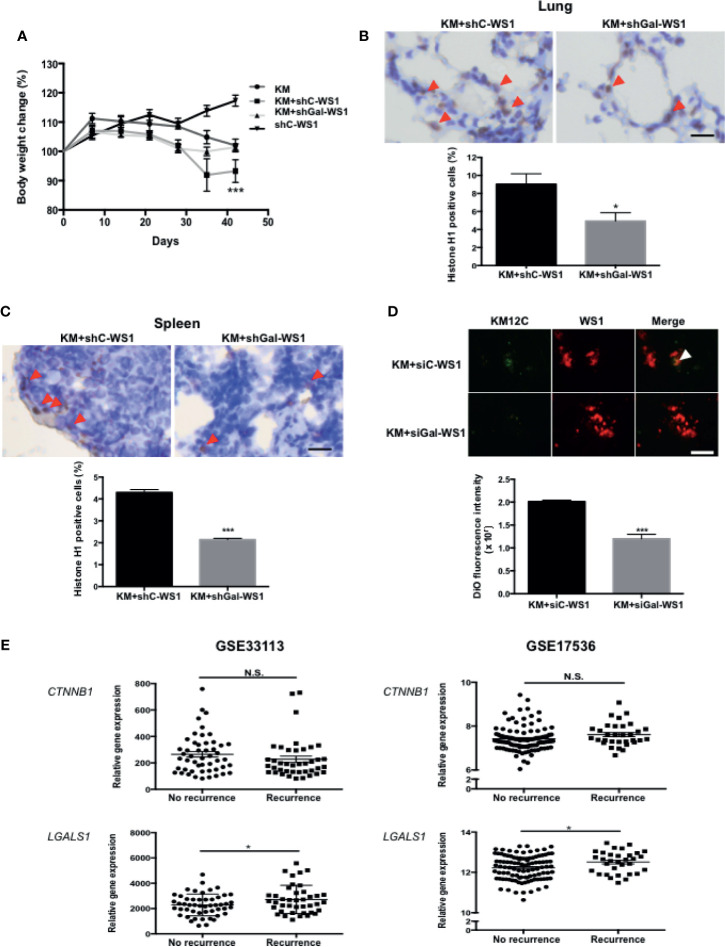
Fibroblast-secreted Gal-1 significantly increases metastasis and tumor dissemination of CRC cells *in vivo*. **(A)** Body weight of NOD-SCID mice 40 days after tail vein injection of KM12C only (3 x 10^5^ cells); KM12C (3 x 10^5^ cells) admixed with WS1 silenced for with short-hairpin RNA (shRNA) of non-target sequences (shC-WS1; 3 x 10^5^ cells); KM12C (3 x 10^5^ cells) admixed with WS1 silenced with shRNA specific for Gal-1 (shGal-WS1; 3 x 10^5^ cells); or shC-WS1 only (3 x 10^5^ cells). Each condition consisted of three mice, with their body weight measured every 7 days. Immunohistochemistry (IHC) staining for human histone H1 (brown nuclei) in **(B)** mouse lung and **(C)** spleen tissue sections; representative sections (top panel) and quantitative results (bottom panel) are shown, with arrows indicating human Histone H1(+) cells; scale bar, 20 μm. **(D)** Visualization of fluorescently labeled co-cultured cells KM12C (3 x 10^5^ cells; green fluorescence, labeled with DiO), and siC- or siGal-WS1 (3 x 10^5^ cells; red fluorescence, labeled with with DiI) in lung sections 24 hours after injection into the tail vein of C57BL/6 mice. Top panel, representative images; bottom panel; quantitative results. Arrows indicate KM12C; scale bar, 100 μm. All results are shown as the mean ± SEM of three independent experiments. *p < 0.05, and ***p < 0.005 compared to the control. **(E)** Analyses of Gal-1 (*LGALS1*) and β-catenin (*CTNNB1*) expression with regard to disease recurrence in the public dataset GSE33113 and GSE17536 of gene expression omnibus (GEO); *p < 0.05; N.S., not significant.

To assess whether the CIC features induced by fibroblast-secreted Gal-1 are involved in CRC disease progression and metastasis, we used two mouse models of metastases to validate our *in vitro* findings: a longer-term metastatic disease model using immunocompromised mice and a rapid lung tumor dissemination model using wild type mice. To assess whether fibroblast-secreted Gal-1 promoted metastatic disease, we generated stable clones of WS1 silenced for Gal-1 expression using short hairpin RNA (shRNA) specific for *LGALS1* (shGal-WS1). We then injected KM12C co-cultured with either shGal-WS1 (KM+shGal-WS1) or with non-target shRNA-silenced WS1 (KM+shC-WS1) into the tail vein of immunocompromised mice for the evaluation of metastatic growths in the lung. As negative and positive controls, we injected mice with WS1 only (shC-WS1; without KM12C) or KM12C only (KM), respectively. After 40 days of follow-up, mice injected with KM+shC-WS1 demonstrated significant decreases in body weight and were nearly moribund when compared to either mice injected with KM+shGal-WS1 or even KM12C only; WS1 only-injected mice, on the other hand, were healthy and demonstrated an increased weight gain over time (**Figure 3A**). Lung and spleen tissue sections from these mice demonstrated a significantly higher number of human cells in the mice injected with KM+shC-WS1 compared to those injected with KM+shGal-WS1 (**Figures 3B, C**; top panel: representative sections, and bottom panel: quantitative results). To ascertain that tumor dissemination was affected by fibroblast-secreted Gal-1, we co-cultured KM12C with either siGal-WS1 (KM+siGal-WS1) or with siC-WS-1 (KM+siC-WS1), and injected cells into the tail vein of C57BL/6 mice which were sacrificed after 24 hours to assess for tumor dissemination within the lungs. Tumor seeding was more significant in the mice injected with KM+siC-WS1 compared with KM+siGal-WS1 (**Figure 3D**). To assess for clinical relevance, we analyzed the human CRC transcriptome databases which contain recurrence information (GSE33113 and GSE17536; **Supplementary Table S2**) and found that high expression levels of *LGALS1*, but not β-catenin (*CTNNB1*), correlate significantly with a high risk of metastasis and/or recurrence within 3 years (**Figure 3E**). Thus, these results demonstrate that fibroblast-secreted Gal-1 significantly promotes metastatic disease progression and tumor dissemination in mouse models, as well as correlate to human CRC disease recurrence.

### Gal-1 Promotes β-catenin Expression, Nuclear Accumulation, and Activity in Colorectal Cancer Cells

**Figure 4:**
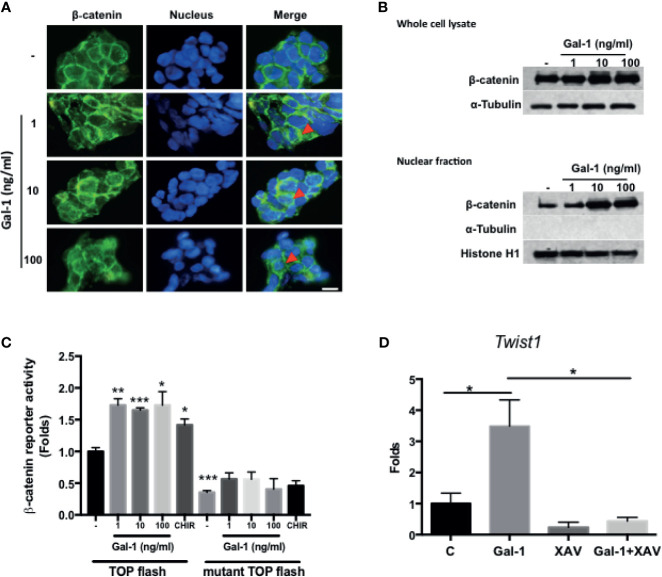
Gal-1 promotes β-catenin expression, nuclear translocation, and activity in CRC cells. **(A)** IF staining for β-catenin (green fluorescence) in KM12C with the addition of increasing doses of rhGal-1 for 48 hours. Nuclei were stained with Hoechst 33342 (blue fluorescence). Arrows show nuclear β-catenin; scale bar, 10 μm. **(B)** Western blot for β-catenin levels in whole cell lysate (top panel) and nuclear fraction (bottom panel) of KM12C with the addition of increasing doses of rhGal-1 for 48 hours; for nuclear fraction, histone H1 is used as the positive control and α-Tubulin as the negative control. **(C)** Luciferase reporter assay for β-catenin activity in KM12C with the addition of increasing doses of rhGal-1. TOPFlash plasmids (β-catenin promoter reporter construct containing TCF/LEF binding sites; please see Materials and Methods) and TOPFlash mutant plasmids (β-catenin promoter reporter construct containing mutated TCF/LEF binding sites; please see Materials and Methods) were transduced into KM12C, with the luciferase activity measured 48 hours later; addition of the Wnt/β-catenin agonist CHIR-99021 (CHIR; 0.3 µM) was used as a positive control. **(D)** qPCR analysis for the gene expression of *Twist1* in KM12C after treatment with rhGal-1 (100 ng/ml) and without or with the Wnt/β-catenin antagonist XAV-939 (XAV; 10 µM) for 24 hours. All results are shown as the mean ± SEM of three independent experiments. *p < 0.05; **p < 0.01, and ***p < 0.005 compared to the control.

Wnt/β-catenin signaling is the central pathway involved in CRC pathogenesis, with the activation of the pathway being a feature of CICs and correlating with a more aggressive disease outcome. We therefore assess whether secreted Gal-1 can activate this pathway in CRC cells. We found that treatment of KM12C with exogenous rhGal-1 induced a cytoplasmic to nuclear translocation of β-catenin, as evidenced by IF staining (**Figure 4A**). This was corroborated by the Western blot data, in which both total as well as nuclear β-catenin levels were increased with increasing doses of rhGal-1 (**Figure 4B**). To further ascertain for the activation of β-catenin activity, we performed luciferase reporter assay by transducing either the wild type or mutant TOPFlash reporter into KM12C and then treating with rhGal-1. We found that all doses of rhGal-1 significantly induce reporter activity over baseline in the wild type but not the mutant promoter (**Figure 4C**). β-catenin has also been found to promote EMT (31), and we found that the addition of rhGal-1 strongly induced the expression of the EMT transcription factor *Twist1* in KM12C, which could be suppressed with XAV-939 (XAV), an inhibitor of the β-catenin pathway (**Figure 4D**). XAV also decreased Gal-1-induced CD29 expression in HCT-116 (**Supplementary Figure S4**). These results therefore demonstrate that secreted Gal-1 could activate β-catenin activity in CRC cells.

### SOX9 Is a Critical Mediator Involved in Gal-1-Induced Upregulation of β-catenin Activity and Cancer-Initiating Cell Features

**Figure 5:**
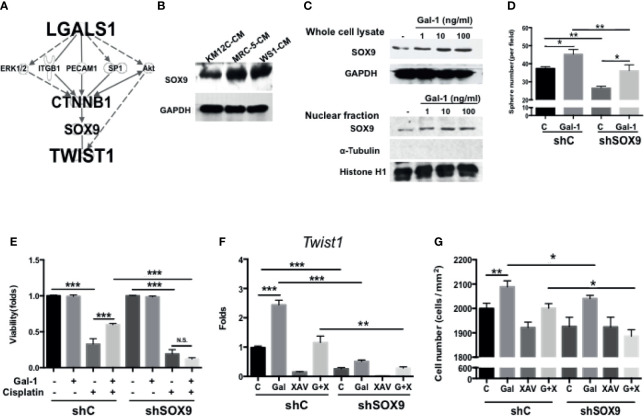
SOX9 is a critical mediator involved in Gal-1-induced upregulation of β-catenin activity and CIC features. **(A)** Ingenuity Pathway Analysis (IPA) for the prediction of candidate mediators within the *LGALS1/CTNNB1/Twist1* axis. IPA database revealed the several major pathways which might be involved in tumor development and metastasis. According to the IPA results and literature review, *SOX9* was selected and confirmed whether it is the downstream gene of Gal-1 by Western blot. **(B)** Western blot for the analysis of SOX9 protein levels in KM12C after culturing in MRC-5- or WS1-CM; KM12C-CM was used as the control. Internal control: GAPDH. **(C)** Western blot for SOX9 levels in whole cell lysate (top panel) and nuclear fraction (bottom panel) of KM12C with addition of increasing doses of rhGal-1 for 48 hours; for nuclear protein blot, histone H1 is used as the positive control and α-Tubulin as the negative control. **(D)** Sphere formation capacity of shC-KM and shSOX9-II-KMC12 (shSOX9-KM) after treating with rhGal-1 (100 ng/ml) for 72 hours. **(E)** Drug resistance capacity of shC- and shSOX9-KM to cisplatin (25 µM) after pretreatment with rhGal-1 (100 ng/ml) for 24 hours. Cell viability was assessed 48 hours after drug treatment. **(F)** qPCR analysis for the gene expression of *Twist1* in shC- and shSOX9-KM after treatment with Gal-1 (100 ng/ml) and XAV (10 µM). **(G)** Invasion capacity of shC- and shSOX9-KM with addition of rhGal-1 (100 ng/ml) and XAV (10 µM). All results are shown as the mean ± SEM of three independent experiments. *p < 0.05; **p < 0.01, and ***p < 0.005 compared to the control. N.S., not significant.

Given the inherent complexity of the Wnt/β-Catenin signaling pathway, we were interested in further elucidating the details in the role of Gal-1 within this critical CRC pathway. To identify/search for other mediators involved in *LGALS1/CTNNB1* interactions, we performed IPA with the addition of TWIST1—a downstream target of β-catenin and one of the most important EMT transcription factors—to the analyses. Using this model, SOX9 was predicted as a key candidate gene within this interaction (**Figure 5A**). The role of this transcription factor in CRC remains unclear despite the very recent large-scale genomic data identifying this gene to be a significant and novel somatic recurrently mutated gene in this cancer (32). We found that the co-culture of both types of fibroblast-CM increased the protein expression of SOX9 in KM12C (**Figure 5B**). Moreover, the addition of rhGal-1 to KM12C not only enhanced the overall SOX9 protein expression levels, but also increased the nuclear levels of the transcription factor (**Figure 5C**). To investigate the role of SOX9 in CIC formation, we generated *SOX9-*silenced KM12C (shSOX9-KM) and confirmed the efficiency of knockdown by Western blot, which identified the shSOX9-II clone as having a more efficient knockdown. Compared to non-target knockdown KM12C (shC-KM), shSOX9-KM had a significantly decreased capacity for sphere formation; moreover, while the addition of rhGal-1 significantly improved the shSOX9-KM sphere formation capacity, this was still significantly less than the capacity of rhGal-1-treated shC-KM (**Figure 5D**). SOX9 also contributes to Gal-1-mediated drug resistance, since we found that shSOX9-KM was significantly more sensitive to cisplatin compared to shC-KM even with rhGal-1 pretreatment (**Figure 5E**; a decrease of 1.00- to 0.12-fold for cell viability in shSOX9-KM compared with 1.00- to 0.60-fold in shC-KM). To assess whether β-catenin was involved in Gal-1/SOX9 interaction, we analyzed for changes in the expression of *Twist1* as a downstream gene of β-catenin in shSOX9- and shC-KM cells with the addition of rhGal-1 and with or without β-Catenin antagonism (**Figure 5F**). We found that the levels of *Twist1* are significantly increased in shC-KM after the addition of rhGal-1, which could then be significantly suppressed to below baseline levels when the β-catenin antagonist XAV was applied; simultaneous addition of rhGal-1 and XAV in shC-KM restored *Twist1* expression to baseline levels. In shSOX9-KM, however, the baseline expression of *Twist1* was lower than the baseline levels in shC-KM; moreover, neither the addition of rhGal-1 nor XAV to shSOX9-KM increased *Twist1* levels. In terms of invasive capacity, migration capacity was decreased in shSOX9-KM compared to shC-KM (**Figure 5G**), but treatment with rhGal-1 increased the migration capacity significantly in shC-KM and non-significantly in shSOX9-KM. Importantly, rhGal-1-induced migration was abrogated after the treatment with XAV in shSOX9-KM but not shC-KM. Together, these results demonstrate that SOX9 is an important mediator involved in Gal-1-induced upregulation of β-catenin signaling activity as well as the augmentation of CIC features in CRC.

### High Expression of Gal-1 and SOX9 Correlate With Clinical Colorectal Cancer (CRC) Outcome

**Figure 6:**
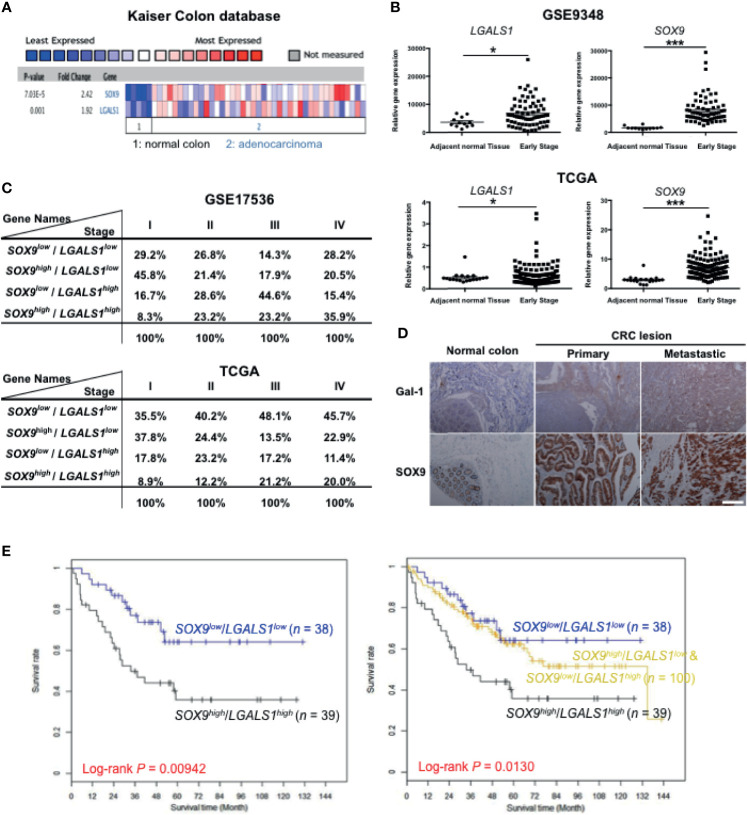
High expression of Gal-1 and SOX9 correlate with clinical CRC outcome. **(A)** ONCOMINE assessment of the expression levels of *LGALS1* and *SOX9* in the Kaiser Colon database. **(B)** Analysis of *LGALS1* or *SOX9* expression levels in tumor tissue compared to adjacent normal tissue using GSE9348 (top panel) and The Cancer Genome Atlas (TCGA) databases (bottom panel). *p < 0.05 and ***p < 0.005 for early-stage lesions compared to adjacent normal tissue. **(C)** Analysis of *LGALS1* and *SOX9* expression levels to the CRC stage using the GSE17536 and TCGA datasets. **(D)** Immunohistological staining of Gal-1 and SOX9 on CRC tumor samples, which included 40 primary lesions (primary), 10 metastatic lesions (metastatic), and 9 normal colon samples; scale bar, 100 μm. **(E)** Kaplan-Meier survival curves of four groups of CRC patients as stratified by median expression levels of SOX9 and Gal-1 in tumor tissue: SOX9^low^/Gal-1^low^, *n* = 38; SOX9^low^/Gal-1^high^ & SOX9^high^/Gal-1^low^, *n* = 100; and SOX9^high^/Gal-1^high^, *n* = 39. Survival analyses was performed for two groups: SOX9^high^/Gal-1^high^ *versus* SOX9^low^/Gal-1^low^ (left-side graph); or for three groups: SOX9^high^/Gal-1^high^ *versus* SOX9^high^/Gal-1^low^ + SOX9^low^/Gal-1^high^ *versus* SOX9^low^/Gal-1^low^ (right-side graph).

**Figure 7:**
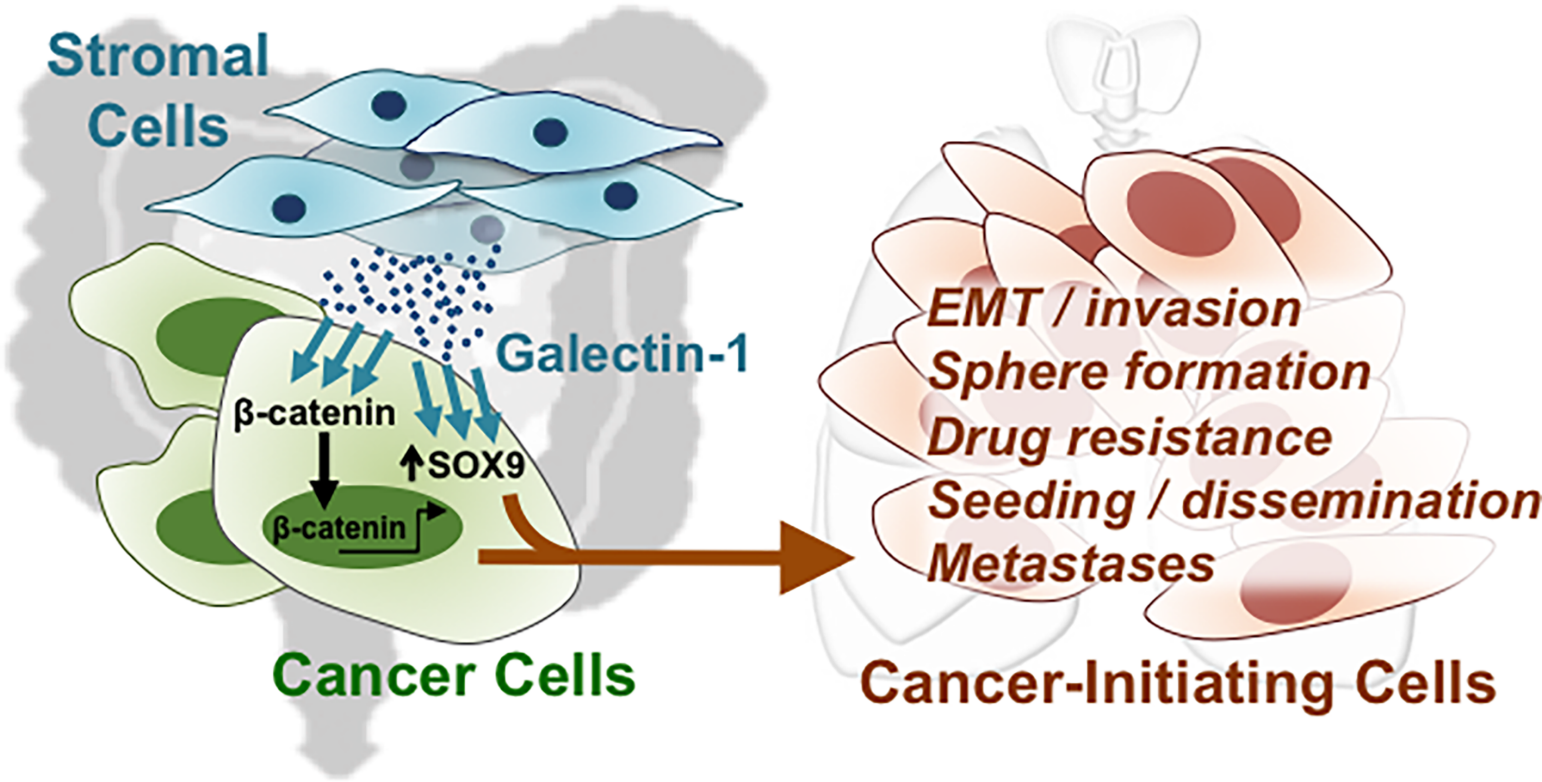
Direct targeting of stromal-secreted Gal-1 on CRC cells promote CIC features and disease progression through SOX9 and β-catenin.

Our results indicate that both Gal-1 and SOX9 promote CIC features, which involve the β-catenin pathway in CRC cells. To assess whether Gal-1 and/or SOX9 expression is clinically relevant for CRC, we analyzed for the expression of either one or both of these genes to various measured clinical parameters in publicly available CRC databases. We first searched the ONCOMINE database of published microarray data with matched normal and cancer specimens, and found, in the Kaiser Colon database, that a higher expression of both *LGALS1* and *SOX9* are seen in CRC samples than in normal colon samples (**Figure 6A**). To further study whether the expression patterns of either or both genes are correlated with more detailed clinical outcomes, we analyzed the gene expression profiles of two datasets from GSE9348 and The Cancer Genome Atlas (TCGA) (**Supplementary Table S2**) which includes early-stage CRC samples and adjacent normal tissue. In both databases, both *LGALS1* and *SOX9* were significantly expressed at higher levels in early-stage CRC tissue compared to adjacent normal tissue, especially *SOX9* (**Figure 6B**). Moreover, in databases with stage-specific gene expression information, such as GSE17536 and TCGA (**Supplementary Table S2**), analyses revealed that the percentage of CRC samples with a high expression of *LGALS1/SOX9 (LGALS1^high^/SOX9^high^*) correlated with an increasing CRC stage (**Figure 6C**): with increasing stage, *LGALS1^high^/SOX9^high^* CRC samples increased from 8.3% to 35.9% and from 8.9% to 20.0% in the GSE17536 and TCGA databases, respectively. On the other hand, neither *CTNNB1/LGALS1* highly expressed (*CTNNB1^high^*/*LGALS1^high^*) nor *CTNNB1^high^*/*SOX9^high^* highly expressed CRC samples correlate with the CRC stage (**Supplementary Figure S5**). To verify the protein expression, we performed IHC of Gal-1 and SOX9 in a human CRC tissue microarray, which included 40 primary lesions, 10 metastatic lesions, and 9 normal colon samples (**Figure 6D**). The tissue array staining revealed that both Gal-1 and SOX9 protein expression were more prominent in CRC samples compared to normal tissue. Furthermore, distinct patterns of Gal-1 *vs*. SOX9 expression within CRC samples could be seen: Gal-1 expression appeared to increase with an increasing disease progression, while SOX9 expression appear to more strongly correlate with the presence of any cancerous lesion. Most critically, survival analyses based on expression levels of SOX9 and Gal-1 demonstrate that CRC patients with SOX9^high^/Gal-1^high^ expression have a significantly shorter survival compared with patients with SOX9^low^/Gal-1^low^ expression (**Figure 6E** and **Supplementary Figure S5**). Collectively, these analyses of clinical data/samples not only demonstrate that Gal-1 and/or SOX9 overexpression strongly correlate with disease presence, but also with the stage of CRC. Moreover, the presence of both Gal-1 and SOX9 together are strong predictors of a worse outcome in terms of disease survival. Along with our *in vitro* and mouse *in vivo* data, these results therefore demonstrate that stromal cell-derived Gal-1 directly target CRC cells to promote CIC features and disease progression through SOX9 and β-catenin (**Figure 7**).

## Discussion

CRC is one of the most common diseases worldwide, and alarmingly in developed nations such as the USA, the incidence and mortality of CRC has begun to increase in younger individuals after decades of decrease for the population at large. While early-stage lesions are amenable to screening and treatment, late and metastatic stage disease still have a dismal 5-year survival of less than 15% (2–5). Thus, there is a critical need for the discovery of biomarkers for early diagnosis as well as relapse. The microenvironment or niche of the solid tumor is increasingly seen to be a crucial partner in mediating disease progression (12, 13, 33–35); we therefore embarked on studying the molecular interactions between tumor niche-stromal fibroblast cells and CRC cells for the discovery of prognostically relevant biomarkers. Our data demonstrate that fibroblast-secreted Gal-1 promotes multiple CIC features in CRC cells including activating β-catenin *in vitro*, promoting metastasis and tumor dissemination *in vivo*, as well as significantly correlating with clinical recurrence and disease progression. These findings strongly suggest that fibroblast-secreted Gal-1 could be involved in promoting the presence of disseminated tumor cells (DTCs), which represent cancer cells that have undergone EMT and can disseminate to distant organ to seed metastatic growths (36, 37). Indeed, we found that fibroblast-secreted Gal-1 enhanced EMT-related gene expression *in vitro* in CRC cells, and increased the number of injected human CRC cells in the lungs in both short-term and long-term *in vivo* mouse models (**Figures 2C, D** and **3C, D**); moreover, high expression of Gal-1 in CRC patients correlated significantly with metastasis and recurrence (**Figure 3E**). Importantly, using transcriptome data and pathway prediction, we found that *SOX9*, a novel CRC driver (32), not only was mechanistically involved in Gal-1/β-catenin interactions but also is a highly relevant biomarker, especially when evaluated in conjunction with Gal-1. These findings collectively demonstrate that tumor niche-derived paracrine factors are not only important in the maintenance of CRC CICs, but can also be prognostically relevant in evaluating clinical disease progression. Our study also outlines the translational utility of niche/non-cancer cell type-based *in vitro* molecular findings to serve as biomarkers, especially given that most patient genomic and transcriptomic databases are derived from bulk tumor specimens that include non-cancer elements of the stroma and immune system.

Gal-1 is a member of the β-galactoside-binding protein family and known to modulate cancer-associated immunosuppression and angiogenesis (14, 20, 38). While the immunomodulatory mechanisms of Gal-1 have been clearly elucidated, its direct role on carcinogenesis has not been studied in much detail even for CRC in which a positive correlation with a worsening disease status has been reported (39, 40). Given the central role of the Wnt/β-catenin pathway in CRC, we postulated that tumor niche-derived paracrine factors might promote disease progression through interactions with this pathway. Our findings on the capacity of Gal-1 to activate β-catenin and induce CRC CIC features not only provide a mechanistic evidence for Gal-1 having direct interactions with CRC cells, but also support previously published clinical correlative findings of Gal-1 to be mainly expressed within the CRC stroma and not the cancer cell itself (38, 39), which we found as well (**Figure 2A**) (16). Critically, our *in vivo* findings strongly support Gal-1 as having an important role in CRC dissemination, which was bore out in analyses of human CRC databases revealing a high Gal-1 expression to be predictive of recurrence. Since Gal-1 is a secreted molecule and released in the circulation, our findings implicate that this marker could be potentially useful as a biomarker in CRC; however, further studies with patient samples are necessary to validate this possibility. Our report therefore provides further molecular understanding on previously reported disease-promoting correlations of Gal-1 in CRC.

We found SOX9, a transcription factor belonging to the *Sry*-related HMG-box family, to be involved in Gal-1/β-catenin interactions in CRC. An important regulator for numerous developmental processes including in the gastrointestinal epithelium (41, 42), SOX9 has been categorized as belonging in the broader Wnt/β-catenin pathway (32). While SOX9 is known to be transcriptionally repressed by β-catenin in cartilage development (43, 44) and found to contribute to a number of cancer types including CRC (15, 45, 46),, only recently through a comprehensive molecular characterization of CRC has mutations in this transcription factor been implicated in any type of human cancers (32). Previous reports on the role of SOX9 in CRC have yielded mixed results, such as a tumor suppressor (47, 48) and having oncogenic functions (15, 49). These conflicting results have been speculated to be due to a complex relationship of SOX9 dosage on function (50). Intriguingly, these studies rarely take into consideration the fact that SOX9 is a key transcription factor in developmental/non-neoplastic EMT processes, as well as in neoplastic disease (51, 52). We found SOX9 to be involved in a Gal-1/β-catenin-mediated enhancement of a number of *in vitro* CIC features including EMT-related gene expression (**Figure 5**); moreover, analyses of multiple human CRC transcriptome databases as well as tissue microarray immunohistological staining demonstrated a significant correlation of high SOX9 expression to tumor presence, which is highly suggestive of its utility as a CRC biomarker. Critically, the simultaneous use of both Gal-1 and SOX9 is strongly correlated with a worse survival in CRC patients (**Figure 6E**). Our findings therefore not only contribute to a molecular understanding on the roles of Gal-1 and SOX9 in the central CRC pathway of Wnt/β-catenin, but also reveal these molecules as useful prognostic markers in transcriptomic databases.

In summary, we found that fibroblast-secreted Gal-1 significantly enhanced multiple *in vitro* CRC CIC properties including enhancing EMT and activating β-catenin, as well as promoting *in vivo* metastatic disease and tumor dissemination, and clinical recurrence. Bioinformatics pathway analyses predicted SOX9, a recently discovered aggressive CRC marker, as being involved in Gal-1/β-catenin interactions, which was validated *in vitro*. Moreover, Gal-1 or SOX9 but not β-catenin are prognostically correlated with disease presence and progression. Critically, a high expression of both Gal-1 and SOX9 is correlated with a significantly worse disease survival. Our findings highlight the critical role of the tumor niche-stromal component of CRC in disease progression and for discovery of prognostic markers and drug targets.

## Data Availability Statement

The datasets presented in this study can be found in online repositories. The names of the repository/repositories and accession number(s) can be found in the article/**Supplementary Material**.

## Ethics Statement

The studies involving human participants were reviewed and approved by the National Health Research Institutes (human cell line use) and GEO and TCGA (public bioinformatics data). The animal study was reviewed and approved by the Institutional Animal Care and Use Committee, NHRI.

## Author Contributions

Conception and design: K-YP and BY. Development of methodology: K-YP, S-SJ, and BY. Acquisition of data (provided animals, acquired and managed patients, provided facilities, etc.): Y-WL and F-YT. Analysis and interpretation of data (e.g., statistical analysis, biostatistics, computational analysis): K-YP, S-SJ, F-YT, C-CC, and BY. Writing, review, and/or revision of the manuscript: K-YP, S-SJ, C-CC, L-TC, and BY. All authors contributed to the article and approved the submitted version.

## Funding 

This study was supported in part by grants from the NHRI (10A1-CSPP06), the Ministry of Health & Welfare (MOHW110-TDU-B-212-144026), and the Taiwan Ministry of Science & Technology (MoST108-2314-B-400-035-MY3 and MoST110-2740-B-400-002).

## Conflict of Interest

The authors declare that the research was conducted in the absence of any commercial or financial relationships that could be construed as a potential conflict of interest.

## Publisher’s Note

All claims expressed in this article are solely those of the authors and do not necessarily represent those of their affiliated organizations, or those of the publisher, the editors and the reviewers. Any product that may be evaluated in this article, or claim that may be made by its manufacturer, is not guaranteed or endorsed by the publisher.

## Supplementary Material

Supplementary Figure 1 Flow cytometric analysis for surface marker expression of CD29 (top panel) and CD44 (bottom panel) in KM12C treated with fibroblast-CM (Conditioned Medium).

Supplementary Figure 2 SDS-PAGE analysis of conditioned media from MRC-5 and WS1.

Supplementary Figure 3 Flow cytometry analysis for surface marker expression of CD29 and CD44 in KM12C (left panel) and HCT-116 (right panel) after Gal-1(100ng/ml) treatment.

Supplementary Figure 4 Flow cytometric analysis for surface marker expression of CD29 in HCT-116 after Gal-1(100ng/ml) and XAV(10uM) treatment.

Supplementary Figure 5 Analyses of the data sets GSE17536 and TCGA for stage-specific expression of *CTNNB1/LGALS1* or *SOX9/CTNNB1*.

Supplementary Table 1 qPCR primer sequence information.

Supplementary Table 2 Information on the public microarray gene expression profiles used within this study.

## Abbreviations

CRC, Colorectal cancer; CICs, Cancer-initiating cells; CM, Conditioned medium; MS, Mass spectrometry; Gal-1, Galectin-1; IPA, Ingenuity pathway analysis; DMEM/F-12, Dulbecco’s Modified Eagle Medium: Nutrient Mixture F-12; FBS, Fetal bovine serum; MEM, Minimum essential medium; qPCR, Quantitative real-time polymerase chain reaction; siRNA, small interference RNA; shRNA, short hairpin RNA; EGF, Epidermal growth factor; bFGF, basic fibroblast growth factor; HRP, Horseradish peroxidase; ELISA, Enzyme linked immunosorbent assay; IF, Immunofluorescence staining; MTT, 3-(4,5-Dimethyl-2-thiazolyl)-2,5-diphenyl-2H-tetrazolium bromide; DiI, 1,1’-Dioctadecyl-3,3,3’,3’-Tetramethylindocarbocyanine Perchlorate; DiO, 3,3’-Dioctadecyloxacarbocyanine Perchlorate; IHC, Immunohistochemistry; OCT, Optimal cutting temperature; GEO, Gene expression omnibus; TCGA, The Cancer Genome Atlas; EMT, Epithelial-mesenchymal transition; rhGal-1, Recombinant human Gal-1 protein; siC-WS1, Non-target siRNA control in WS1; siGal-WS1, siRNA specific for Gal-1-knockdown in WS1; shC-WS1, Non-target shRNA control in WS1; shGal-WS1, shRNA specific for Gal-1-knockdown in WS1; KM, KM12C; XAV: XAV939; shC-KM, Non-target shRNA control in KM12C; shSOX9-KM, shRNA specific for SOX9- knockdown in KM12C; DTCs, Disseminated tumor cells.
