# Supplementary figures and images for: Stromal Galectin-1 Promotes Colorectal Cancer Cancer-Initiating Cell Features and Disease Dissemination Through SOX9 and β-Catenin: Development of Niche-Based Biomarkers
Source: Front Oncol. 2021 Sep 10;11:716055. doi: 10.3389/fonc.2021.716055 (PMC8462299; doi:10.3389/fonc.2021.716055)

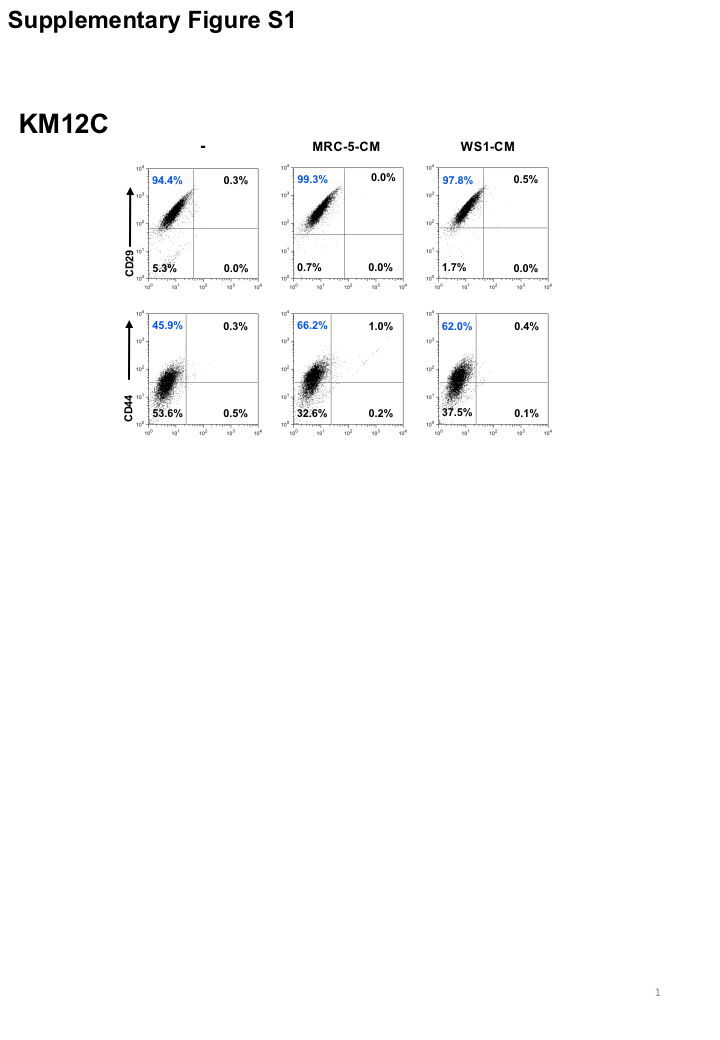

Supplement: Supplementary Figure 1 — Flow cytometric analysis for surface marker expression of CD29 (top panel) and CD44 (bottom panel) in KM12C treated with fibroblast-CM (Conditioned Medium). [file Image_1.tiff]

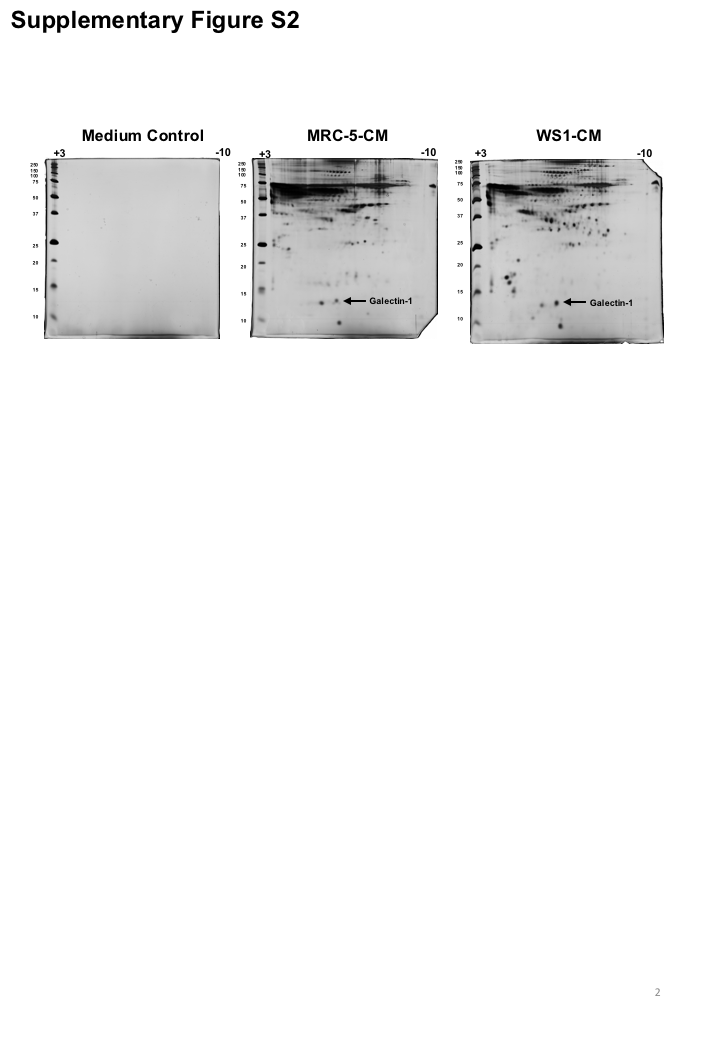

Supplement: Supplementary Figure 2 — SDS-PAGE analysis of conditioned media from MRC-5 and WS1. [file Image_2.tiff]

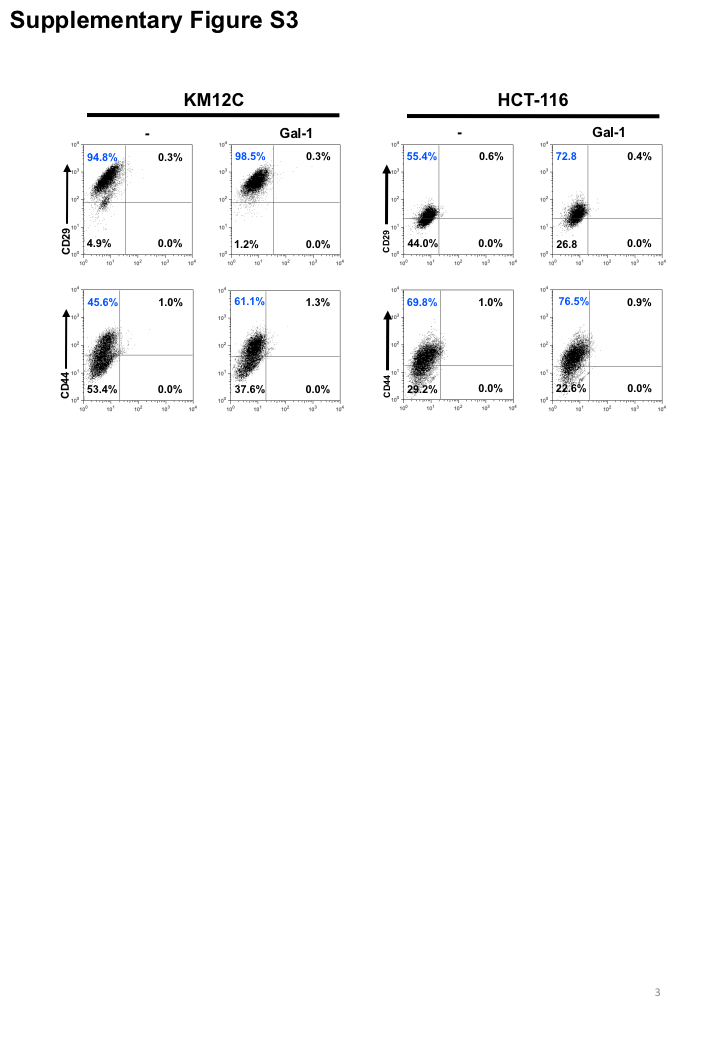

Supplement: Supplementary Figure 3 — Flow cytometry analysis for surface marker expression of CD29 and CD44 in KM12C (left panel) and HCT-116 (right panel) after Gal-1(100ng/ml) treatment. [file Image_3.tiff]

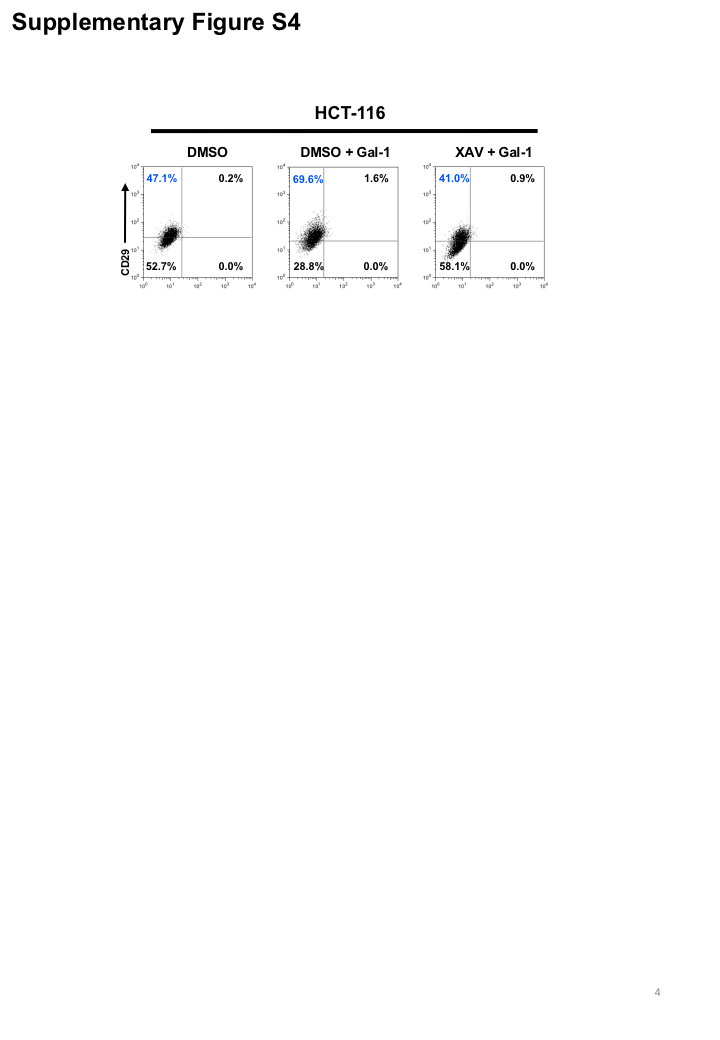

Supplement: Supplementary Figure 4 — Flow cytometric analysis for surface marker expression of CD29 in HCT-116 after Gal-1(100ng/ml) and XAV(10uM) treatment. [file Image_4.tiff]

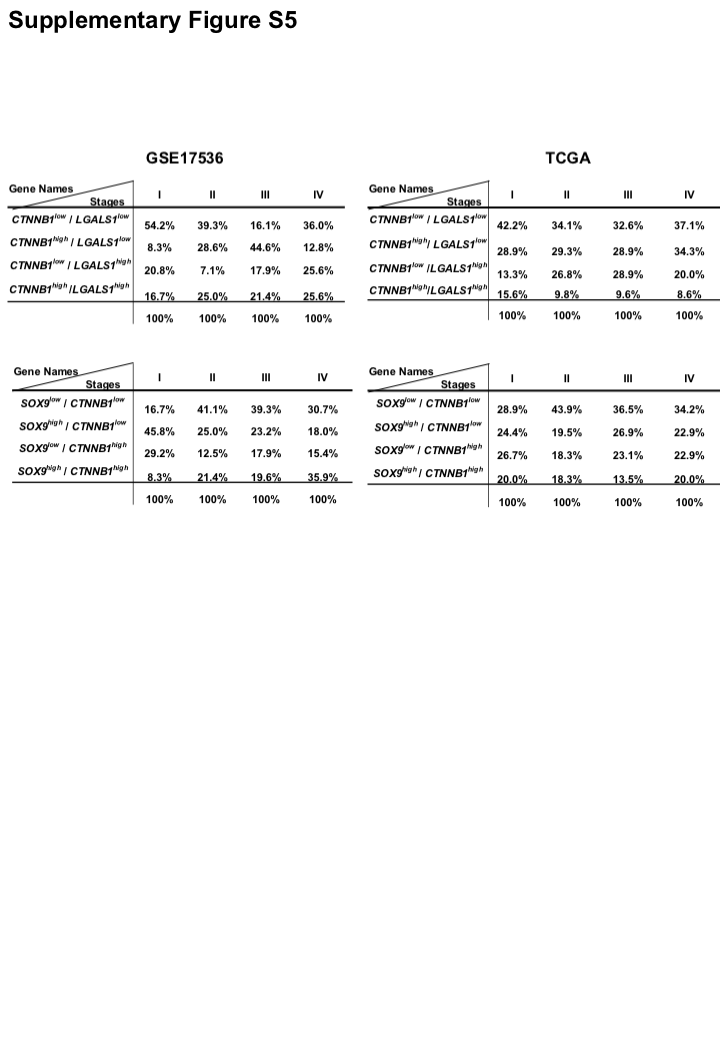

Supplement: Supplementary Figure 5 — Analyses of the data sets GSE17536 and TCGA for stage-specific expression of CTNNB1/LGALS1 or SOX9/CTNNB1. [file Image_5.tiff]

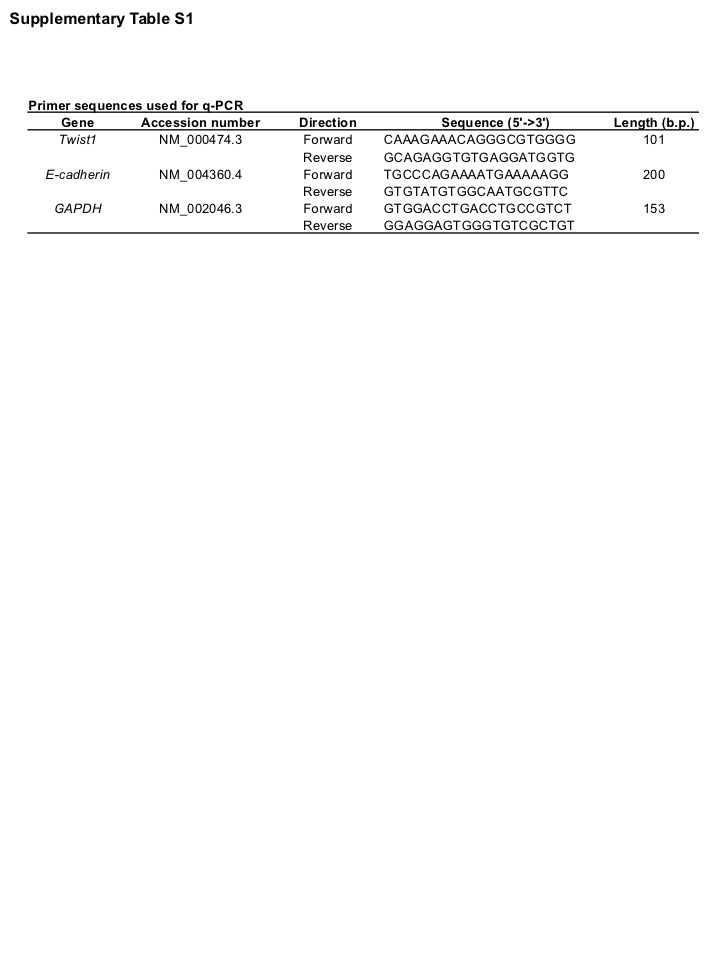

Supplement: Supplementary Table 1 — qPCR primer sequence information. [file Image_6.tiff]

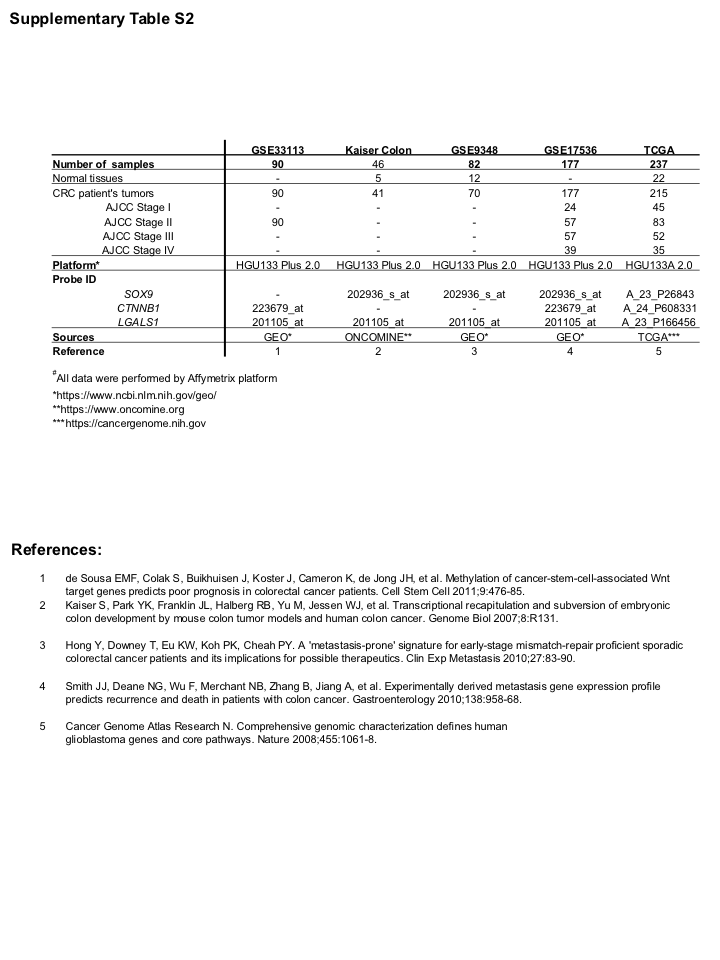

Supplement: Supplementary Table 2 — Information on the public microarray gene expression profiles used within this study. [file Image_7.tiff]
